# Characterizing the spatial and temporal variation of malaria incidence in Bangladesh, 2007

**DOI:** 10.1186/1475-2875-11-170

**Published:** 2012-05-21

**Authors:** Heidi L Reid, Ubydul Haque, Shyamal Roy, Nazrul Islam, Archie CA Clements

**Affiliations:** 1 Infectious Disease Epidemiology Unit, Level 4 Public Health Building, School of Population Health, University of Queensland, Herston, QLD, 4006, Australia; 2 Department of Molecular Microbiology and Immunology, Johns Hopkins Bloomberg School of Public Health, Baltimore, MD, 21205, USA; 3 Ministry of Health and Family Welfare, Malaria and Parasitic Disease Control, Director General of Health Services, Mohakhali, Dhaka, Bangladesh

**Keywords:** Malaria, Bangladesh, Spatial, Temporal, Seasonality, Bayesian, *Plasmodium falciparum*, *Plasmodium vivax*

## Abstract

**Background:**

Malaria remains a significant health problem in Bangladesh affecting 13 of 64 districts. The risk of malaria is variable across the endemic areas and throughout the year. A better understanding of the spatial and temporal patterns in malaria risk and the determinants driving the variation are crucial for the appropriate targeting of interventions under the National Malaria Control and Prevention Programme.

**Methods:**

Numbers of *Plasmodium falciparum* and *Plasmodium vivax* malaria cases reported by month in 2007, across the 70 endemic *thanas* (sub-districts) in Bangladesh, were assembled from health centre surveillance reports. Bayesian Poisson regression models of incidence were constructed, with fixed effects for monthly rainfall, maximum temperature and elevation, and random effects for *thanas*, with a conditional autoregressive prior spatial structure.

**Results:**

The annual incidence of reported cases was 34.0 and 9.6 cases/10,000 population for *P. falciparum* and *P. vivax* respectively and the population of the 70 malaria-endemic *thanas* was approximately 13.5 million in 2007. Incidence of reported cases for both types of malaria was highest in the mountainous south-east of the country (the Chittagong Hill Tracts). Models revealed statistically significant positive associations between the incidence of reported *P. vivax* and *P. falciparum* cases and rainfall and maximum temperature.

**Conclusions:**

The risk of *P. falciparum* and *P. vivax* was spatially variable across the endemic *thanas* of Bangladesh and also highly seasonal, suggesting that interventions should be targeted and timed according to the risk profile of the endemic areas. Rainfall, temperature and elevation are major factors driving the spatiotemporal patterns of malaria in Bangladesh.

## Background

Spatial and temporal variability in malaria transmission are known to be driven partly by ecological factors that affect the survival and population size of anopheline vectors. Temperature, humidity and water available for breeding habitats have been shown to be important primary ecological factors [1]. Fluctuation in malaria risk has significant public health implications for ensuring adequate provision of anti-malarials, the delivery of intermittent preventative therapy (IPT) and optimizing the timing and frequency of indoor residual spraying with insecticides and/or distribution of long-lasting insecticidal nets (LLIN), and thus warrants investigation.

At a macro-scale, attempts have been made to understand the mechanisms driving malaria risk. The Mapping Malaria Risk in Africa (MARA) collaboration developed maps that describe the expected duration and timing of the transmission season by country based on climate suitability models [2,3]. Insight into the underlying ecological mechanisms both inhibiting and propelling malaria transmission has led to research into malaria early warning systems (MEWS) to be able to predict disease patterns based on known relationships between the disease and ecological variables [4-11].

A host of issues, however, make characterizing the natural phenomena underlying spatial and temporal patterns in malaria risk difficult. Differences in ecological requirements between malaria vectors mean that particular environmental events, such as the rainy season, can lead to an increase in vector capacity for most vectors but an initial decrease for others [12-14]. Variability due to ecological drivers can be further complicated by patterns in host immunity, which is a possible explanation for intra-annual and inter-annual variation [15-17]. In addition, the longitudinal datasets required for analysis of both spatial and temporal patterns are commonly obtained from national health information system systems, from which the accuracy of estimates is highly variable [18].

The advancement of geographical information systems (GIS) and spatial statistics has greatly improved the understanding of malaria dynamics, including its dependence on ecological factors. Bayesian geostatistical models are now commonly employed to generate malaria risk maps providing a valuable evidence base for programmatic decision-making [19-26]. For malaria data aggregated by administrative area, as is often the case with health information system data, Bayesian conditional autoregressive (CAR) models have been used to model spatial and temporal patterns [17,27-29]. This approach has the advantage of accounting for spatial correlation in the data and smoothing out variability associated with small populations in some areas.

Malaria remains a significant health problem in Bangladesh affecting 13 of 64 districts. In 2006, the Global Fund to Fight AIDS, Tuberculosis, and Malaria awarded Bangladesh $39.6 million US dollars to support the national malaria-control programme [30]. Three key objectives of the programme are to provide effective diagnosis and treatment to 80% of estimated malaria cases by 2012, to promote the use of LLINs in 80% of households in these districts, and to use selective indoor residual spraying for containment of outbreaks [30].

While spatial patterns of *Plasmodium falciparum* prevalence have been previously described in Bangladesh [26], spatiotemporal patterns of *P. falciparum* and *Plasmodium vivax* incidence have not. The aims of this study were to describe the spatiotemporal patterns of reported malaria cases and identify environmental drivers of the spatiotemporal patterns of malaria risk in Bangladesh. This information may affect the timing and geographical targeting of interventions such as indoor residual spraying and distribution of LLINs. Through the more focussed implementation of interventions, the findings presented here have the potential to enhance the effectiveness of the national malaria control programme and provide valuable baseline epidemiological information upon which to chart the progress of the programme.

## Methods

### Malaria surveillance data and descriptive methods

The Bangladesh Ministry of Health lists malaria as a notifiable disease and maintains routine surveillance of clinical malaria nationwide. Cases of malaria diagnosed at health facilities are reported to the *thana* health complex, and collated up through the district and division administrative levels. From 2004 to 2009, cases in Bangladesh were confirmed as uncomplicated malaria presumptive (UMP), uncomplicated malaria confirmed (UMC), as well as severe malaria (SM) and vivax malaria (VM). Neither microscopy nor rapid diagnosis tests (RDT) were performed for UMP, but for the others, either microscopy or a RDT was used for diagnosis. According to official national figures, 98% of confirmed cases were diagnosed using microscopy in 2007 and 2% using a RDT, with approximately 76% of cases being UMP [31]. Since 2007, the situation has changed, with 63% of cases diagnosed using a RDT and only 10% of cases being UMP in 2010 [32].

For the purposes of the present study, reported numbers of *P. vivax* and *P. falciparum* malaria cases across all age groups were assembled by month for each of the 70 *thanas* in the 13 endemic districts between January and December 2007. Population data from the national census conducted in 2001 were adjusted according to national growth figures [33], assuming an even population growth across all *thanas*. The population was then used as a denominator to calculate malaria incidence in each *thana*. In the 13 malaria-endemic districts, the incidence of reported *P. falciparum* malaria cases for the whole of 2007 was compared to prevalence determined by population-based surveys using RDTs conducted in the same year [34]. Pairwise, Pearson cross correlations between incidence of *P. falciparum* and *P. vivax* for each month (January–December) were calculated to determine whether the distribution of the two species was related.

### Climate and elevation data

A number of ecological and climatic factors affect both the extrinsic life cycle of the malaria parasite and that of the anopheline vectors [35]. Based on published literature, variables pertinent to malaria transmission in Bangladesh were selected for statistical analysis. High-resolution (1 sq km) raster maps of interpolated long-term average monthly rainfall, and minimum and maximum temperature, were obtained from the WORLDCLIM project [36]. Digital elevation data (in meters above sea level for cells of a 1 sq km grid), were obtained from the same source.

Data concerning vegetation cover were obtained from the GlobCover Land Cover product, which is derived from satellite imagery from 2005 to 2006 [37]. Vegetation was labelled according to the United Nations (UN) Land Cover Classification System [38] at a resolution of 300 m. For this analysis, vegetation cover was dichotomized into forested, which included multiple specific forest categories, and not forested, which included all other vegetation categories.

Rainfall, temperature, elevation and vegetation maps were imported into the geographical information system (GIS) ArcView version 9 (ESRI, Redlands, CA, USA) and linked spatially to a digitized boundary map of the 70 malaria-endemic *thanas*. For each *thana*, the mean values of the raster cells contained in the *thana* for rainfall, temperature and elevation, and percentage forest cover, were computed in the GIS to define covariates in subsequent models. Environmental variables were standardized by centreing the covariates on their mean and dividing by the standard deviation to assist mixing and convergence of the spatiotemporal models.

### Statistical analysis

Bayesian spatiotemporal Poisson regression models were constructed using the WinBUGS software, version 1.4.3 (MRC Biostatistics Unit, Cambridge, UK). The outcome was the monthly number of reported cases of *P. vivax* or *P. falciparum* malaria (January to December 2007) in each *thana* and the offset was the expected number of cases based on the population of each *thana*.

The final regression model was:

(1) $$O_{\mathrm{ij}}\sim Poisson(\mu_{\mathrm{ij}})\text{,}$$

where *O*_*ij*_ is the observed number of cases in *thana i*, month *j* and

(2) $$\log(\mu_{\mathrm{ij}})=\log(E_{i})+\theta_{\mathrm{ij}}\text{,}$$

where *E*_*i*_ is the expected monthly number of cases in *thana i*, which did not vary by month because the population was considered static. The mean log relative risk was modelled as:

(3) $$\theta_{\mathrm{ij}}=\alpha +\beta \times elev_{i}+\delta \times prec_{\mathrm{ij}}+\varphi \times temp_{\mathrm{ij}}+s_{i}$$

where *α* is the intercept, *βδ* and ∅ are the coefficients for mean elevation (*elev*), precipitation (*prec*), and maximum temperature (*temp*), respectively, and *s*_*i*_ is a spatial county-level random effect. Note, minimum temperature and percent forest cover were excluded because of collinearity (Pearson’s correlation > |0.7|) with maximum temperature and elevation, respectively. Spatial structuring in *s*_*i*_ was modelled using a conditional autoregressive prior structure, where spatial relationships between counties were modelled using a simple adjacency weights matrix [39]. If two counties were adjacent, the weight = 1 and if they were not adjacent, the weight = 0. Non-informative priors were used for the intercept (an unbounded uniform, or flat, prior), coefficients (Gaussian priors with mean = 0 and variance = 10,000) and the variance of *s*_*i*_ (a gamma prior with shape and scale parameters = 0.01). Models were created with, and without, the environmental covariates to determine their ability to capture spatial and temporal effects in the data. The residuals of the models were examined to determine whether any temporal patterns, including seasonality and temporal autocorrelation, remained after adjusting for the effects of rainfall and temperature. Additionally, models were compared using the deviance information criterion (DIC) to determine whether incorporation of the environmental covariates improved the fit of the models (a lower DIC indicates a better-fitting model).

## Results

**Figure 1:**
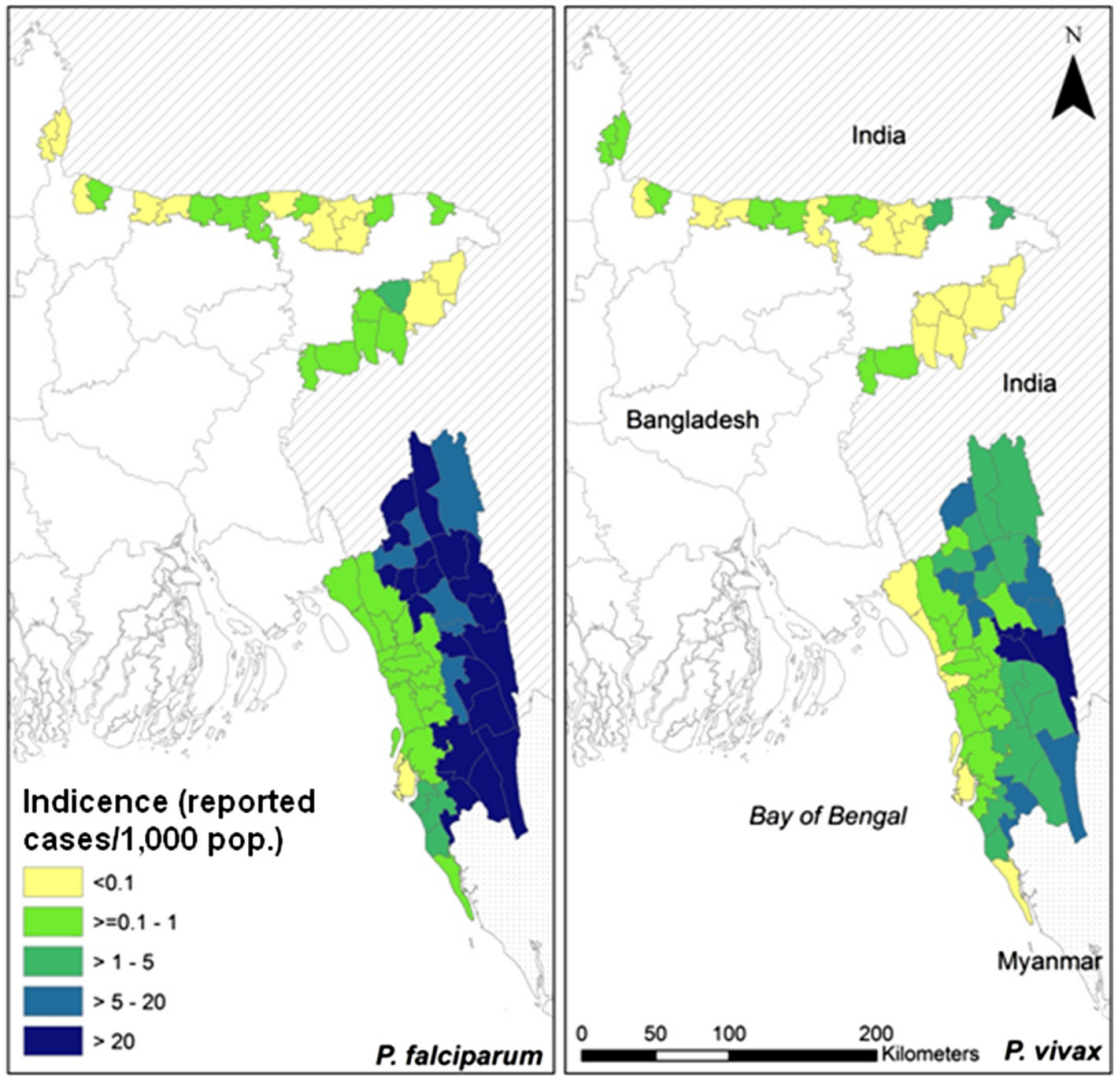
**Incidence of reported cases of *Plasmodium falciparum* and *Plasmodium vivax* per 1,000 people across the endemic *thanas* in Bangladesh, 2007.**

The population of the 70 malaria-endemic *thanas* was approximately 13.5 million in 2007. In these *thanas*, there was a total of 45,761 reported cases of *P. falciparum* malaria in 2007, representing a crude incidence of 34.0 cases per 10,000 people; and 12,968 reported cases of *P. vivax* malaria, representing a crude incidence of 9.6 cases per 10,000 people. Maps revealed high reported incidence of both species in the south-east of the country (i.e. the Chittagong Hill Tracts) (Figure 1).

**Figure 2:**
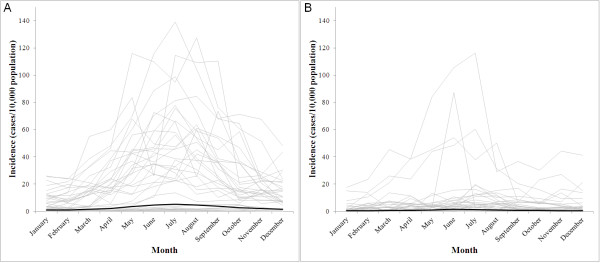
** *Thana* -level monthly incidence (grey lines) and overall reported incidence across endemic *thanas* (black lines) of reported cases of A.***Plasmodium falciparum* and B. *Plasmodium vivax* malaria in Bangladesh, 2007.

**Figure 3:**
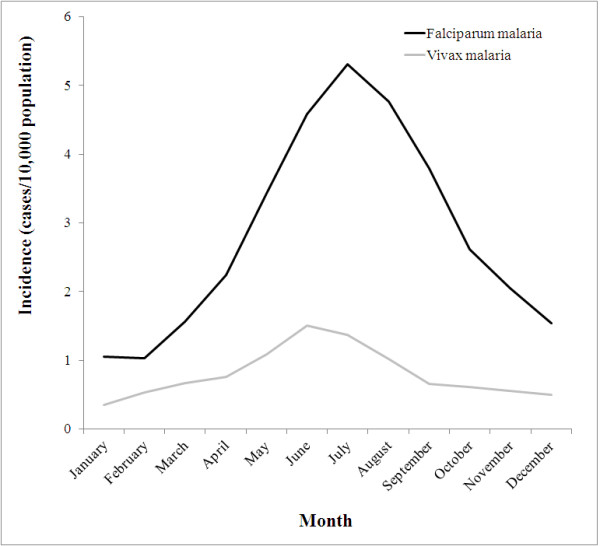
**Monthly incidence of reported cases of *Plasmodium falciparum* and *Plasmodium vivax* across endemic *thanas* in Bangladesh, 2007.**

**Figure 4:**
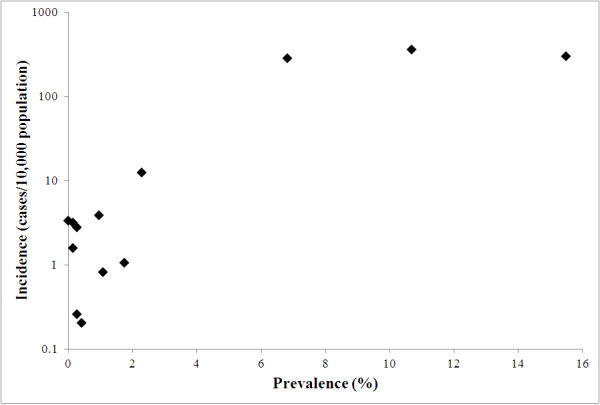
**A comparison of incidence of reported cases of *Plasmodium falciparum* malaria by district and the prevalence of *P. falciparum* infection from population-based surveys using rapid diagnostic tests in the 13 malaria-endemic districts of Bangladesh, 2007.**

The monthly incidence of reported *P. vivax* and *P. falciparum* cases is shown by *thana* in Figure 2. It is evident that in some *thanas* there was a strong seasonal pattern but that incidence was highly over-dispersed. Seasonal comparisons between the two types of malaria are clearer on a plot of the overall incidence for the 13 endemic districts (Figure 3). *P. falciparum* showed a peak in incidence in July while *P. vivax* peaked in June. There was a moderately high correlation (range 0.30–0.62) between incidence of reported *P. falciparum* and *P. vivax* in any given month (Table 1). At the district level, there was also a strong positive association between the incidence of reported cases of *P. falciparum* malaria and the prevalence of *P. falciparum* infection determined by the population-based surveys conducted in the same year (Figure 4).

**Table 1:** **Pearson cross correlations comparing the *thana* -level incidence of *Plasmodium vivax* with *Plasmodium falciparum* for each month**

**Month**	**Correlation between the incidence of *P. falciparum* and *P. vivax* in each month**
	*P. falciparum*/*P. vivax*
January	0.52
February	0.52
March	0.36
April	0.45
May	0.30
June	0.30
July	0.38
August	0.62
September	0.50
October	0.39
November	0.39
December	0.55

The *P. falciparum* and *P. vivax* models revealed similar positive associations with maximum temperature and monthly precipitation (Tables 2 and 3). The impact of elevation on the risk of *P. falciparum* and *P. vivax* differed, with a higher risk of *P. falciparum* and a lower risk of *P. vivax* at increasing elevation.

**Table 2:** **Bayesian Poisson regression models of *Plasmodium falciparum* , Bangladesh, 2007**

**Variable**	**With environmental covariates**	**Without environmental covariates**
Intercept	−1.23 (−1.28 - -1.19)	−1.02 (−1.06 - -0.98)
Elevation (10 m increase)	1.45 (1.39–1.49)	
Maximum temperature (1°C increase)	1.012 (1.011–1.013)	
Precipitation (100 mm increase)	1.15 (1.14–1.15)	
Variance CAR random effect (*s*_*i*_)	0.10 (0.07–0.14)	0.15 (0.10 – 0.21)
Deviance Information Criterion	11774.6	23356.4

*Estimates for the covariates are relative risks; results show mean and 95% credible interval (CrI).

**Table 3:** **Bayesian Poisson regression models of *Plasmodium vivax* , Bangladesh, 2007**

**Variable**	**With environmental covariates**	**Without environmental covariates**
Intercept	−1.02 (−1.14 - -0.92)	−0.93 (−1.05 - -0.84)
Elevation (10 m increase)	0.98 (0.96 – 1.00)	
Maximum temperature (1°C increase)	1.010 (1.009 – 1.011)	
Precipitation (100 mm increase)	1.09 (1.09 – 1.10)	
Variance CAR random effect (*s*_*i*_)	0.11 (0.07–0.15)	0.12 (0.08 – 0.17)
Deviance Information Criterion	8950.1	10735.8

Residual analysis indicated that incorporation of environmental variables was effective in removing seasonality from the data, with only a small amount of unstructured temporal variation and no temporal autocorrelation evident in the residuals (indicating that it was not necessary to incorporate a temporal autoregressive term in the model). The environmental covariates were also able to explain some of the spatial variability in malaria counts, measured through the variance of the spatial random effect (*s*_*i*_). Inclusion of environmental variables into the models reduced the variance of the spatial random effect by 32.4% for *P. falciparum* and 6.7% for *P. vivax* compared to models without environmental covariates (Table 2). Incorporation of the environmental models improved the fit of the models for both types of malaria, as indicated by a lower DIC.

## Discussion

Malaria in Bangladesh exhibits distinct spatial and temporal patterns, with varying levels of endemicity and significant, biologically tenable relationships to environmental variables. Importantly, the analysis provides insight into the relatively unexplored epidemiology of *P. vivax* within the country.

The inclusion of ecological covariates in the model provided some explanation as to the temporal and spatial variation in malaria risk. Temperature and rainfall showed an expected positive relationship with increased malaria risk, which is consistent with similar analyses of incidence data from Ethiopia, Peru and China [17,40,41]. These two variables were effective in explaining the seasonal patterns in the data. The environmental covariates could explain a sizable percentage of spatial variability observed in distribution of *P. falciparum* but only a relatively modest percentage seen with *P. vivax*. Other unmeasured factors including local malaria control activities [42], socio economic indicators [43] and population movement [44] are likely to contribute to variability in malaria risk, to differing degrees for the two species. The positive relationship between *P. falciparum* and elevation (after adjusting for the effects of temperature) could be explained by higher elevations providing denser forest cover and a more favourable environment for forest breeding vectors known to sustain transmission in these areas [45]. However, given that the vectors for the two species overlap in Bangladesh, the opposite finding for *P. vivax* was unexpected. With little known about the epidemiology of *P. vivax* in the country, this finding requires further investigation. However, of note, the odds ratio for elevation in the *P. vivax* model was of a small magnitude and was only marginally significant.

*Plasmodium falciparum* and *P. vivax* showed broadly similar spatial distributions which is consistent with more recent entomological information that suggests the same vectors are able to transmit both species. A study conducted in three ecologically distinct areas of the Chittagong Hill Tracts identified eight *Anopheles* species to be positive for circumsporozoite protein with a number of these vectors infected by both *P. falciparum* and *P. vivax *[46]. Prior to this study no data were available documenting vectors of *P. vivax* in Bangladesh. The moderately high correlation between the incidence of *P. falciparum* and *P. vivax* for each of the months supports the entomological evidence, although this could be affected by factors such as geographical differences in the quality of laboratory practices that are used to differentiate malaria due to the different parasites. The correlation between *P. falciparum* and *P. vivax*, and the effect estimates in the *P. vivax* models, could also be sensitive to relapsing of vivax malaria. Unfortunately the surveillance system was not able to differentiate relapsing infections from primary *P. vivax* infections and it was not possible to test the potential impact of relapsing infections. Inter-species interactions, including cross-protective immunity, might influence observed heterogeneity of malaria infections in areas co-endemic for *P. falciparum* and *P. vivax*[47]. Additionally, background or ongoing malaria control could influence the ability to accurately quantify correlations between *P. falciparum* and *P. vivax* malaria and effect estimates (such as between elevation and malaria incidence) in the analysis.

The results presented here suggest that *P. vivax* is more widespread than found in cross-sectional surveys conducted in the same year [34]. Depending on the accuracy of diagnosis of *P. vivax* malaria in the surveillance system, this has significant implications for treatment of *P. vivax*, with national treatment guidelines recommending the use of chloroquine and primaquine [48]. Primaquine has been associated with acute haemolytic anaemia in patients with G6PD deficiency and, therefore, knowledge of the prevalence and geographical distribution of this inherited blood condition in *P. vivax* endemic areas is important [49]. The only cross-sectional data on G6PD deficiency in Bangladesh found a prevalence of 10.7% [50].

An important consideration for this analysis is that the data were obtained through the national passive surveillance system, which is known to grossly under-report malaria infections [34]. One contributing factor is that many infections are asymptomatic. Asymptomatic infections have been shown to be significant in Bangladesh, with two to 40 asymptomatic cases per 1,000 population reported in the Chittagong region [51]. Additionally, cases who seek treatment through private health services are not registered by the national surveillance system. Misdiagnosis and under-reporting of malaria infections might affect the estimate of mean incidence, the proportion of cases that are *P. vivax* and the observed spatial patterns in the data, if there was systematic spatial variation in the quality of the surveillance system, as well as the effect estimates of the environmental covariates. Without being able to measure the quality of the surveillance system, it is not possible to speculate as to what the magnitude of these effects might be. However, the comparison of incidence reported through the national passive surveillance system, and prevalence determined by population-based survey methods, showed strong congruence at the district level (Figure 4). This is consistent with recent research that indicates incidence (calculated according to active case detection data) increases smoothly and then flattens out as prevalence increases [52], increasing confidence in the observed spatial patterns in the incidence data reported here. Seasonal migration of people, particularly workers, might also affect observed spatial patterns. Another limitation is the short time series (12 months) used in this analysis. A longer time series is needed to establish longer-term temporal variability of malaria, including important inter-annual patterns, seasonal patterns, and the predictive value of climatic risk factors.

Health information system data have been used to establish spatial variation of disease risk for *P. falciparum* and *P. vivax* in Bangladesh. Given the observed spatial and temporal variation, targeting of interventions has the potential to enhance the effectiveness of the national malaria-control programme in Bangladesh.

## Competing interests

The authors declare that they have no competing interests.

## Authors' contributions

HLR, UH and ACAC conceived the study. HLR conducted the analysis and drafted the manuscript. UH compiled the data, assisted with the analysis and interpretation of the results, and contributed to the final draft. SR and NI facilitated access to the data and provided contextual information necessary to interpret the findings. ACAC provided guidance on the analysis and drafting of the manuscript. All authors read and approved the final manuscript.
